# Physiological Conjunction of Allelochemicals and Desert Plants

**DOI:** 10.1371/journal.pone.0081580

**Published:** 2013-12-10

**Authors:** Avital Yosef Friedjung, Sikander Pal Choudhary, Nativ Dudai, Shimon Rachmilevitch

**Affiliations:** 1 Jacob Blaustein Institutes for Desert Research, French Associates Institute for Agriculture and Biotechnology of Drylands, Ben-Gurion University of the Negev, Sede Boqer Campus, Midreshet Ben Gurion, Israel; 2 The Unit of Medicinal and Aromatic Plants, Agricultural Research Organization, Newe Ya’ar Research Center, Ramat Yishay, Israel; 3 Department of Botany, University of Jammu, Jammu, India; Centro de Investigación y de Estudios Avanzados, Mexico

## Abstract

Plants exchange signals with other physical and biological entities in their habitat, a form of communication termed allelopathy. The underlying principles of allelopathy and secondary-metabolite production are still poorly understood, especially in desert plants. The coordination and role of secondary metabolites were examined as a cause of allelopathy in plants thriving under arid and semiarid soil conditions. Desert plant species, *Origanum dayi*, *Artemisia sieberi* and *Artemisia judaica* from two different sources (cultivar cuttings and wild seeds) were studied in their natural habitats. Growth rate, relative water content, osmotic potential, photochemical efficiency, volatile composition and vital factors of allelopathy were analyzed at regular intervals along four seasons with winter showing optimum soil water content and summer showing water deficit conditions. A comprehensive analysis of the volatile composition of the leaves, ambient air and soil in the biological niche of the plants under study was carried out to determine the effects of soil water conditions and sample plants on the surrounding flora. Significant morpho-physiological changes were observed across the seasons and along different soil water content. Metabolic analysis showed that water deficit was the key for driving selective metabolomic shifts. *A*. *judaica* showed the least metabolic shifts, while *A*. *sieberi* showed the highest shifts. All the species exhibited high allelopathic effects; *A. judaica* displayed relatively higher growth-inhibition effects, while *O. dayi* showed comparatively higher germination-inhibition effects in germination assays. The current study may help in understanding plant behavior, mechanisms underlying secondary-metabolite production in water deficit conditions and metabolite-physiological interrelationship with allelopathy in desert plants, and can help cull economic benefits from the produced volatiles.

## Introduction

Allelopathy is a widely documented phenomenon occurring in natural and man-made ecosystems in which plants release natural products (allelochemicals) that influence the establishment and growth of neighboring plants [1], [2]. Alleopathy has been mostly studied in terms of correlative evidence based on the identification of allelochemicals being released in potent concentrations from leaves, roots and stems [1], [3], [4]. However, due to the complexity of the chemicals, it is difficult to determine the precise role of a specific natural compound in allelopathy [5]. A large variety of natural compounds are known to cause allelopathy, with secondary metabolites constituting the most important group of allelochemicals [5]. Most allelopathy experiments are based on isolating putative compounds and testing their phytotoxicity in vitro. However, most plant interactions are mediated in soil environments; therefore the inclusion of soil as a testing ground for the determination of allelopathic interactions is warranted [1], [6]. Furthermore, an influence of soil behavior on allelochemical activity cannot be ruled out, as several allelochemicals have shown a decline in potency when applied in soil suspensions vs. solution. Thus, the reported role of soil in reducing the phytotoxicity of natural products again suggests its inclusion as a platform to study allelopathic interactions among plants [6]–[8]. Allelochemicals are usually produced in plant cells and accumulate in specific organs, sometimes in special organelles. Leaves may be the most consistent source, while stems and roots are considered to contain less potent toxins [8], [9]. Allelochemicals are released by plants into the soil or atmosphere by volatilization or leaching from the aerial plant parts, eventually becoming deposited on other plants or soils. Leaching may also occur through plant residues, exudation from plant roots into the soil environment and decomposition of plant residues, releasing toxic substances [6]–[11].

In general, allelochemicals are representing a myriad of chemical compounds from simple hydrocarbons and aliphatic acids to complex polycyclic structures [6]–[9]. Allelochemicals include simple water-soluble organic acids and unsaturated lactones, long-chain fatty acids and polyacetylenes, naphthoquinone, anthroquinones and complex quinones, simple phenols, benzoic acid and derivatives, cinnamic acid and derivatives, flavonoids, tannins, terpenoids and steroids, amino acids and polypeptides, alkaloids and cyanohydrins, sulfides and glucosides, purines and nucleotides, coumarins, thiocyanates, lactones and actogenins [8]. Allelochemicals can act indirectly through alteration of soil properties, nutritional status, population composition or activity of microorganisms and nematodes [2]. They can also act directly via biochemical/physiological effects on various important processes of plant growth and metabolism, such as mineral uptake, mitosis (inhibition), hormonal regulation, respiration (stimulation or inhibition), protein synthesis (inhibition) [8], enzyme activity (inhibition) [12]–[13], membrane permeability [2], photosynthesis (inhibition) [14] and more.

In agriculture, the effect of weeds on crops, crops on weeds and crops on crops have been thoroughly studied [2]. Weeds interfere with crops by inhibiting their germination and seedling establishment [15]–[17]. Plants thriving in arid and semiarid regions of the world are known to face water deficit conditions. Several phenotypic adaptations and acclimations such as reduced plant growth, modified leaves into spines, phylloclades and cladodes, and metabolic alterations in metabolisms of sugars, amino acids, plant hormones and large number of secondary metabolites have been documented in desert plants [9], [18]–[19]. However, the role of allelopathy or secondary metabolites in overcoming arid conditions, in conjunction with their allelopathic behavior, is still poorly understood. On the other hand, our current understanding of allelopathy has been successfully used to control weed populations via methods involving crop rotation, mixed cropping and essential oils [20].

The concept of allelopathy is still a matter of controversy and its study is plagued with methodological problems, particularly regarding the distinction between allelopathy and competition [18]–[21]. An extensive literature survey shows a paucity of information on the role of allelochemicals in plant survival in arid and semiarid conditions in combination with allelopathy. The present study focused on three wild perennial species: *Origanum dayi post*, *Artemisia sieberi* and *Artemisia judaica,* growing in arid and semiarid regions of Israel.

Among these three wild species, *O. dayi* is a small shrub that is endemic to Israel, growing in the northern and western Negev desert [22] and in the Judea desert at altitudes of 300 m below sea level to 600 m above sea level [23]. *O. dayi* was domesticated and a selected cultivar is grown as an agricultural crop in Kibbutz Urim in the northern Negev for essential-oil production [24], [25].

*A. sieberi* is a perennial small shrub of Irano-Turanian phytogeographical origin with a wide distribution in the Mediterranean and Middle East, particularly Iran, Israel, Palestine, Syria, Iraq, Turkey, Afghanistan, Spain and Cyprus and extending into central Asia up to the Himalayan Mountains [26]. Essential oil and secondary metabolites obtained from *A. sieberi* have antioxidant, anti-venom, anti-bacterial, anti-spasmodic, neurological and hypoglycemic activities, exhibit cytotoxicity and gene induction, and have toxic effects on reproduction as well as pesticidal activity [26]. Allelopathic effects of *A. sieberi* have a strong impact on plant growth in the wild and on seed germination in vivo [27], and its essential oil has been used in biological weed control.

*A. judaica* is a perennial shrub with small silver-like leaves and bright yellow flowers that appears in March–April. It grows in desert wadi beds in the eastern Sahara–Libya and Egypt, and in the southwestern regions of the Middle East–Sinai, Israel, Jordan and Saudi Arabia. In Israel, *A. judaica* can be found along the Paaran wadi and in the Arava valley from altitudes of 500 m above sea level to 340 m below sea level. In Sinai, the plants can be found at altitudes ranging from 1,600 m above sea level down to the coastal plains [28]. Similar to some other desert *Artemisia* species [29], *A. judaica* is strongly aromatic, and is commonly used by the local population for medicinal purposes, such as for gastrointestinal disorders, snake and scorpion bites, ear infections, dysentery, coughing and external wounds [28]. The Israeli population of *A. judaica* was separated from the Sinai population, creating two distinct chemotypes which reflect genetic differences [30]. Furthermore, the Negev chemotype is of the artemisyl-oil type, characterized by the existence of artemisyl-skeleton-type compounds in the essential oil; the Sinai chemotype is of the piperitone-oil type, characterized by the absence of artemisyl-skeleton-type compounds and the presence of a relatively high percentage of piperitone and camphor [28], [30], [31]–[32]. In addition, it has been reported that the essential oil of *A. judaica* can strongly inhibit the germination of annual plants [33], suggesting use of these allelochemicals for weed control in agriculture.

The mechanisms underlying the survival of desert plants under harsh conditions is not fully known; the present study attempts to elucidate the role of allelochemicals in these species’ survival, and the allelopathic effects of natural compounds released from these species on neighboring plants growing under arid and semiarid conditions. Knowledge gained from the current study will be helpful in understanding the role of allelopathy in desert plants. To explore its role in plant survival under arid and semiarid conditions, extensive morphological, physiological, metabolomic and allelopathic analyses were carried out. The four (summer, spring, winter and autumn) seasons were surveyed separately to study the participation of allelochemicals in allelopathy and survival of desert plants.

## Materials and Methods

### Plant Materials

Three wild species–*Origanum dayi* Post (family Lamiaceae), *Artemisia sieberi* and *Artemisia judaica* (family Compositae)–were used in the present study.

### Growth Conditions

The plants were grown in a greenhouse under standard conditions for 3 months (February to May 2009). The conditions in the greenhouse were: temperature held constant at 20°C (day) and 15°C (night) and relative air humidity kept at 70%. Plants were arranged randomly in different pots in the greenhouse. After 3 months, they were transplanted into the field. From September 2009, the field was divided into nine plots and irrigation regime was calculated by the “Penman equation” using data from the meteorological station located in Sede-Boker:

The irrigation amount was calculated by multiplying the weekly average evaporated water by a crop factor which was correlated to the amount of plant cover. The optimal plant nutrition was supplied equally to all treatments by applying compost at the beginning of the growing season and slow-release fertilizer (Multigan N:P:K 20∶11∶16 Ekogan®) at the base of the plants once every four months. The water conditions in the soil were maintained similar to produce natural conditions having high water content in winter seasons and low water in the summer season as per data provided by meteorological station located in Sede-Boker.

### Physiological Parameters

The following plant physiological stress indicators were measured once a season (four times in total) during the experimental period (autumn–September 2009, winter–January 2010, spring–April 2010 and summer–July 2010).

#### Growth rate

Growth rate was measured by foliage maximum and minimum diameter and plant’s maximum height. The measurement was taken in six replicates–each replicate sampled from a different plant from the seedling and cutting populations.

#### Leaf relative water content (RWC)

Fresh leaf samples (3–10 g) were weighed and soaked in tap water for 24 h until fully turgid. Samples were then reweighed (turgid weight), dried for 48 h at 65°C and weighed again after removal from the oven.

The measurements were taken in six replicates with each replicate sampled from a different plant from the cutting population.

#### Osmolarity

Small segments of fully expanded leaves were macerated in liquid nitrogen and ground into a fine powder. Then 200 µl of double-distilled water was mixed with the crushed leaves to make liquid extract, followed by centrifugation for 1 min at 7500 g. Then, 10 µl of centrifugate was utilized to determine the osmolarity in a pressure osmometer (Vapro® 5520, Wescor, CITY, STATE, USA).

#### Soil water content

Soil water content was measured in two seasons (winter and summer) reflecting significant changes in soil among seasons. The measurements were carried out twice: winter (January 2010) and summer (July 2010). In brief, about 500 g of soil was collected from rhizospheric zone of each plot in four locations: near the plant base on the surface and at 20 cm depth, and 40 cm from the plant base on the surface and at 20 cm depth in respective seasons. Water content was determined by weighing a sample of the soil (fresh weight), oven-drying for 48 h at 65°C and then reweighing (dry weight).

#### Photochemical activity

A portable fluorometer (pulse-amplitude-modulated photosynthesis yield analyzer, Mini-PAM; Walz GmbH, Effeltrich, Germany) was used to measure chlorophyll fluorescence of the leaves in the field. This instrument was used to measure effective quantum yield (Δ*F*/*F*_m’_), calculated as the ratio of F_v_/F_m_, where *F* is the fluorescence yield of the light-adapted sample and *F*_m_(maximum fluorescence) is the maximum light-adapted fluorescence yield when a saturating light pulse of 800 ms duration (intensity = 3,000 µmol m^−2^ s^−1^) is superimposed on the prevailing environmental light levels. The measurements were tested in six replicates–each replicate was sampled from a different plant from the cutting population.

### Determination of Volatile Composition

For the determination of the volatile composition, leaves positioned in the middle of the plant stem and soil samples taken from the rhizosphere zone of the plants, 5 cm belowground were collected and extracted with either organic solvents or water.

#### Extractions in organic solvent

Samples of the leaves or soils were extracted in hermetically sealed 20-ml scintillation vials for 24 h by gentle shaking with methyl tert-butyl ether (MTBE) (99.8%) (Bio-Lab, Israel) containing 10 µg ml^−1^ isobutyl benzene (Sigma-Aldrich, Israel) as a internal standard at room temperature [22]. The sample-to-solvent ratio was 1 g per 10 ml in the case of plant extraction and 2 g per 10 ml in the case of soil extraction. The extracts were purified by passing through a Pasteur pipette containing anhydrous sodium sulfate (Merck, Israel) and Silica gel 60 (230–400 mesh; Merck, Israel) to dry and filter the water and other polar compounds with high molecular weight (such as chlorophyll) that might interfere with the GC-MS analyses. Purified samples were collected into 2-ml Teflon-covered glass vials and injected into the GC-MS system for analysis. The measurement was performed in six replicates, with each replicate consisting of samples from a different plant population–both cuttings and seedlings–in the autumn (September), and only from the cutting population in the winter (January), spring (April) and summer (July).

#### Hydrodistillation

Fresh leaf samples (250 g) were hydrodistilled for 1.5 h in a modified Clevenger apparatus at Newe Ya’ar Research Center as described previously [22], [34]. The essential oil was cooled, separated from the water and analyzed by GC-MS.

#### Solid-phase microextraction (SPME)

SPME was used to ascertain the effect of the plants under study on the surrounding atmospheric and soil conditions. About 20 ml of open vial, 15 vials for each species, were exposed for 6 h, from 0800 to 1400 h, in the summer in the open air, 5 cm aboveground at the plant base. The vials were then sealed and SPME injection needle was inserted manually through the septum into the vial. The headspace volatiles were adsorbed onto the polydimethylsiloxane-divinylbenzene-coated 65 mm SPME fiber for 30 min at ambient temperature and desorbed for 10 min into the GC-MSD (Agilent Technologies, Palo Alto, CA, USA) apparatus injector liner.

Separation and identification of volatile components was performed by GC-MS at the Newe Ya’ar Research Center. The SPME samples or 1 µl of the extract or diluted distillate was analyzed in a GC-MSD equipped with a mass-selection −5973 network (electron ionization 70 eV) detector (Agilent Technologies). The GC-MS was equipped with a capillary column, Rtx-5Sil MS (Restek Corporation, State College, PA, USA) (30 m×0.25 mm) i.d×0.25 µm silica. The carrier gas was helium (He) at a constant flow of 1 ml min^−1^. The extraction samples were introduced into the column in ‘splitless’ mode, while the oil and SPME samples were introduced in ‘split’ mode at a ratio of 1∶50. Temperatures were set as follows: injector temperature 250°C, transfer line and detector 280°C. The column temperature gradient was set as follows: 50°C for 1 min, addition of 5°C min^−1^ to 260°C and held at 260°C for 10 min. Component recognition was based on a comparison of the components’ retention time index (RI) to commercial standards and of the samples’ mass spectrum with GC-MS libraries (Adams 2001, NIST 98, and QuadLib 1607).

### Determination of Allelopathic Effects

#### Allelopathic effects in the field

Allelopathic effects on the weed growth in the field were monitored in the spring by systematically identifying weeds, counting the number of weeds in the different botanical families, collecting them, oven-drying them for 48 h at 105°C and weighing the total biomass. A second round of weed monitoring was carried out in the summer of 2010 when only total biomass was measured.

#### Allelopathic effects of the essential oil: in-vitro experiment

Germination inhibition by different amounts of essential oil was examined in the lab under controlled conditions. Wheat seeds (*Triticum aestivum* L.) were germinated in 20- ml scintillation vials on three layers of Whatman no. 1 filter paper wetted with 1.5 ml distilled water. Vials containing 10 seeds were incubated in the dark at 27°C. To determine the inhibitory effect of an essential oil, five different essential oil quantities: 0.5, 1, 1.5 and 2 µl, as well as blank controls (no essential oil) were tested. The essential oil, as described in [22], was loaded (using a calibrated glass microcapillary) on a piece of filter paper attached by double-sided adhesive tape to the inner side of the vial cover. Each amount of essential oil was tested in five replicates. After incubation, the number of germinated seeds was counted.

#### Allelopathic effects of the soil: in-vivo experiment

This was performed following the methods described in [35] and [36]. In brief, wheat seeds were germinated in 6-cm diameter petri dishes containing 4 g of soil taken from the field. The soil was collected from the upper 1 cm layer, within a 10-cm radius of the plant base, from three well-irrigated plots–one of each species. About 4 ml of distilled water was added and 10 seeds were placed on the wet soil. The petri dishes were incubated in the dark at 27°C for 48 h. As a control, soil from an empty plot in the field was used. Each treatment was tested in five replicates. After incubation, the numbers of germinated seeds were counted.

### Principal Component Analyses (PCA)

PCA is widely used to determine the effects of variables (heat, drought, salinity and other abiotic or biotic stresses) on other selected variables (plant height, yield and the like). In the present investigation, PCA was used to determine the effects of seasonal variations on metabolite production in *O. dayi*, *A. sieberi* and *A. judaica*. Extensive application of PCA can help elucidate the impact of seasonal changes on metabolite production and can also reveal the water-stress-tolerance potential of the species under study. In addition, PCA can be a useful way of describing the significant changes in metabolite production in the different seasons.

### Statistical Analysis

Analysis of variance (ANOVA) was performed using the JMP 7.0.1 statistical package (SAS, 2007). Tukey’s HSD test and Student’s *t*-test were performed to determine possible statistically significant differences between means. The significance level was set at *P*<0.05.

## Results

### Soil Water Content and Plant Height

**Figure 1:**
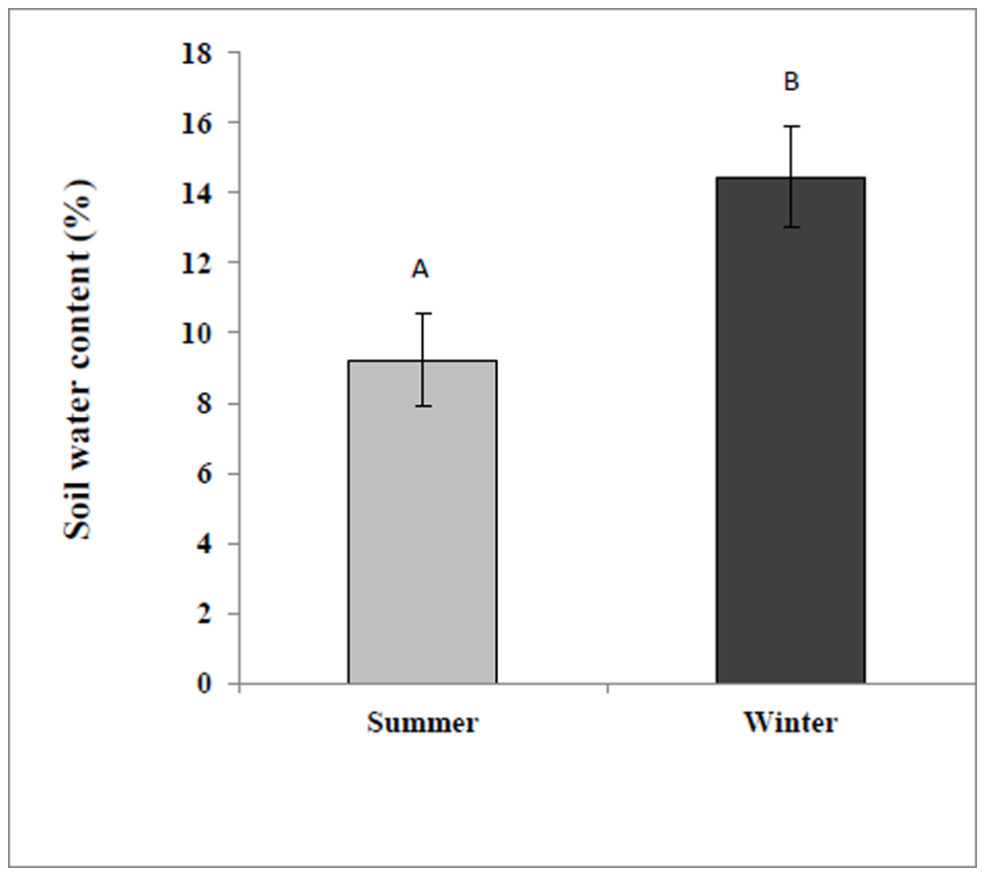
Soil water content as a function of seasonal variations, showing changes between summer (gray) and winter (black), n = 6. Error bars represent standard error. Uppercase letters represent significant difference (P<0.05).

The irrigation treatments were given only for a period of less than two months i.e. from the beginning of the September 2009, to end of October, 2009 (autumn) of all the plots (**Figure 1**). The winter rains started at the end of the October and lasted until the end of March 2010. During this span of five months rainy season the amount of precipitation in the study area–Sede Boker–was 154.6 mm (source Sede-Boker Meteorological station), which was over 50% of the annual mean precipitation. The raining season was followed by a non-raining summer of 2010 (five months of dry period) producing drought conditions in the soil. The field capacity includes a volumetric water content of 25%. We observed a significant decrease (P<0.05) in water content of the soil among summer and winter seasons (**Figure 1** **)**, such that it was significantly lower in summer. Water contents of in the summer and winter were 64 and 40% lower that field capacity.

### Morpho-physiological Parameters

#### Effect of seasonal variations on growth pattern

**Figure 2:**
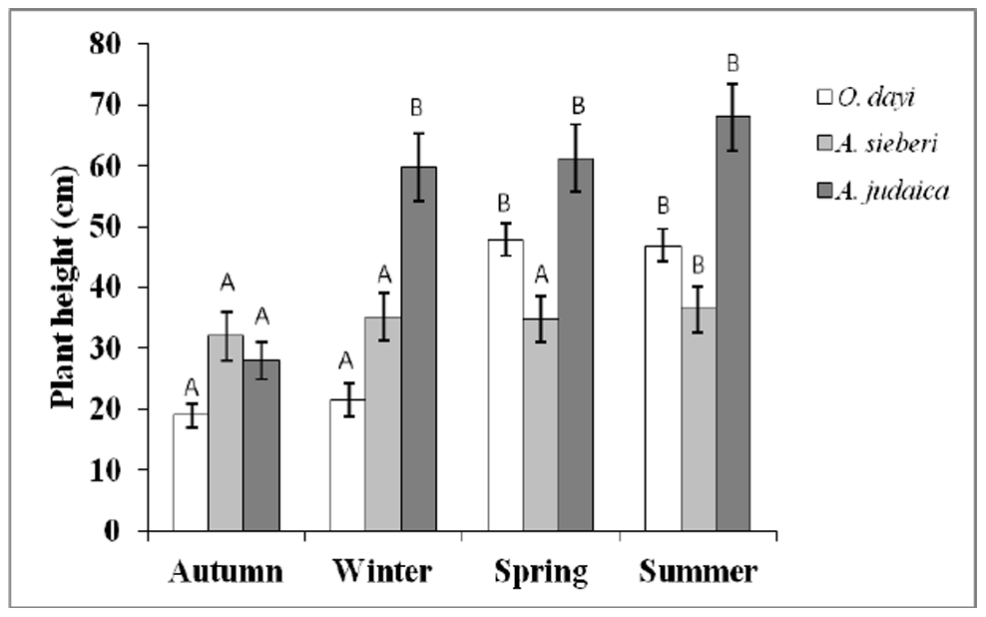
*O. dayi* (white bars), *A. sieberi* (light gray bars) and *A. judaica* (dark gray bars) plant height in the different seasons, n = 5–6. Error bars represent standard deviation. Different uppercase letters represent significant differences within seasons (P<0.05).

Plant growth is a vital indicator of overall plant performance under hosting environments. In the present study, growth was measured in terms of height and diameter of the whole plant. For *O. dayi*, major plant growth was measured in the period between winter (average height of 19.8 cm, average diameter of 41.5 cm) and spring (average height of 44.1 cm, average diameter of 71.3 cm) (**Figures 2** **,** **3**). In contrast, *A. sieberi* showed significant differences (*P*<0.05) in growth between the autumn (average height of 31.6 cm, average diameter of 44.2 cm) and summer (average height of 36.3 cm, average diameter of 51.1 cm) (**Figures 2** **,** **3**). For *A. judaica*, significant differences (*P*<0.05) were observed between the autumn and all other seasons, which did not differ from each other. The main growth occurred in the period between autumn (average height of 38.5 cm, average diameter of 66.9 cm) and winter (average height of 59.9 cm, average diameter of 128.4 cm) (**Figures 2** **,** **3**).

**Figure 3:**
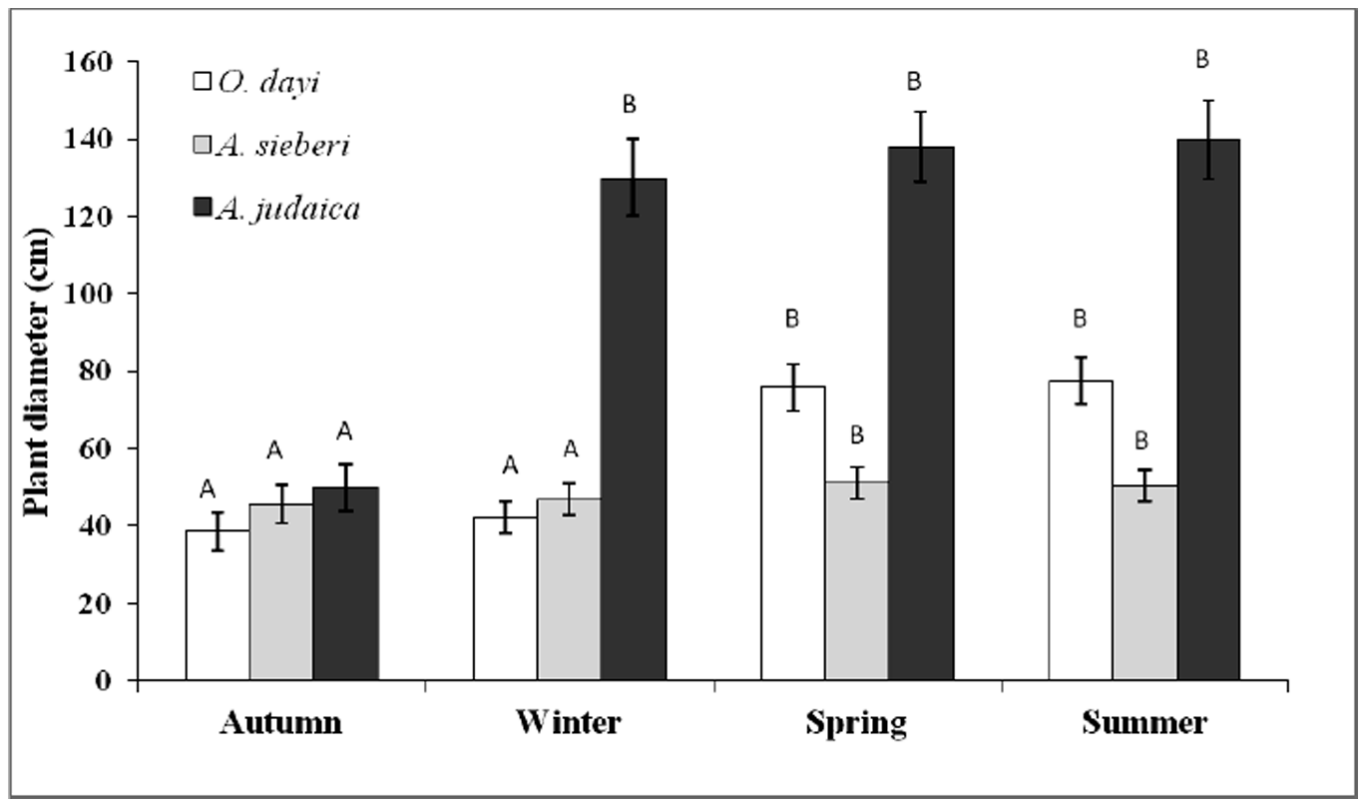
*O. dayi* (white bars), *A. sieberi* (light gray bars) and *A. judaica* (dark gray bars) plant diameter in the different seasons, n = 5–6. Error bars represent standard deviation. Different uppercase letters represent significant differences within seasons (P<0.05).

#### Leaf RWC

For *O. dayi*, a comparison between the seasons showed significant differences (*P*<0.05), with highest RWC values measured in the winter (88.3%), and lowest in the summer (53%) (**Figure S1**). For *A. sieberi*, signficant difference in RWC values were measured in summer period, similarly in winter RWC values were significantly lower 22.2% as compared to an average value of 61.3% in other seasons (**Figure S1)**. RWC measurements for *A. judaica* showed seasonal differences (*P*<0.05), with highest RWC values measured in the winter (average of 76.7%) and lowest RWC in the autumn (average of 53.6%) (**Figure S1**).

#### Osmolarity

For *O. dayi*, there were no significant differences between the spring and summer seasons (*P*>0.05). Interestingly, the autumn and winter seasons showed significant differences in osmolarity (*P*<0.05), but with no consistent trends. A comparison between the seasons showed significant differences (*P*<0.05) in the autumn relative to the spring and summer seasons from 252.2 mmol kg^−1^ to values of 508.8 mmol kg^−1^ in the autumn and summer, respectively (**Figure S2**). Insignificant differences in osmolarity were found for *A. sieberi* between the seasons (*P*>0.05). However, osmolarity values in summer (502.3 mmol kg^−1^) were higher than in autumn (436 mmol kg^−1^) (**Figure S2**). In *A. judaica,* there were significant differences (*P*<0.05) in osmolarity in the autumn and summer (average 349 mmol kg^−1^) relative to the winter and spring (average 441.4 mmol kg^−1^) (**Figure S2**).

#### Photochemical activity

For *O. dayi*, comparison between the seasons showed significant differences (*P*<0.05) between the summer (average value of 0.8) and autumn and winter (average value of 0.77) (**Figure S3**). In *A. judaica*, there were significant differences (*P*<0.05) between the summer (average value of 0.79) and autumn and winter (average value of 0.75) (**Figure S3**).

### Effect of Seasonal Regimes on Volatile Composition

#### Effect of extraction methods on the composition of volatile components

**Figure 4:**
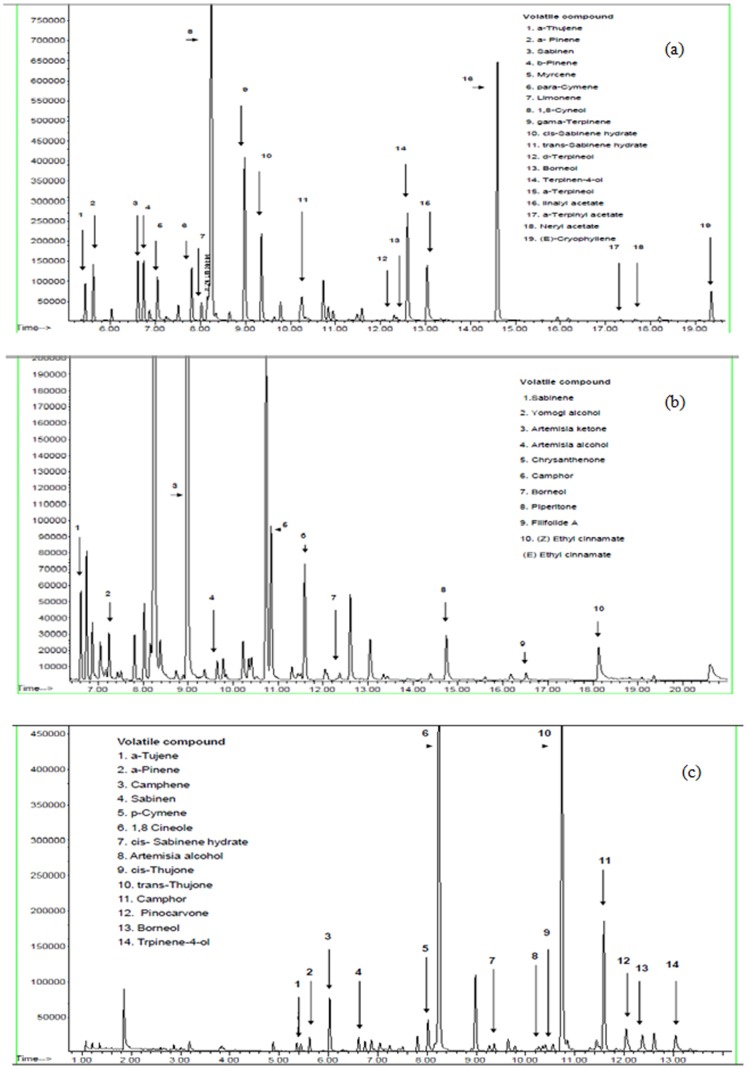
Solid-phase microextraction (SPME) chromatogram of samples taken from the air surrounding *O. dayi* (A), *A. sieberi* (B) and *A. judaica* (C) plants with peaks corresponding to identified metabolites.

In *O. dayi*, about 26 main volatile components were characterized using the organic solvent extraction method (constituting 87.7% of total volatiles), whereas the hydrodistilled sample showed 19 volatile components (**Table 1** **,** **Figure 4A**). In *A. sieberi*, about 21 major components (selected from 83 components and constituting 94.7% of the total volatiles) were characterized by organic solvent extraction vs. 18 components in the essential oil (**Table 2** **,** **Figure 4B**). In *A. judaica*, about 18 components (selected from 63 components and constituting 75.6% of the total volatiles) were detected, as compared to 15 components in the essential oil (**Table 3** **,** **Figure 4C**). In terms of relative differences in the contents of components identified by the solvent- vs. water-extraction methods, large differences were found for *O. dayi* in the relative contents of the components present in the essential oil as compared to organic solvent extraction, for example para-cymene (14.5 and 3.5%, respectively), terpinen-4-ol (18.2 and 4.5%, respectively), α-terpineol (24.7 and 6.6%, respectively), sabinene (4.7 and 8.1%, respectively), cis-sabinene hydrate (4.3 and 13.1%, respectively), Δ-pinene (4 and 10.2%, respectively) and α-terpinyl propionate (0 and 5.1%, respectively) (**Table 1** **,** **Figure 4A**). Similarly in *A. sieberi*, camphor (31.4 and 8%, respectively), 1,8-cineole (21.6 and 9.5%, respectively), para-cymene (8.2 and 0.5%, respectively), trans-thujone (4.1 and 1.6%, respectively), borneol (13.6 and 5.1%, respectively) and germacrene D (1 and 4%, respectively) showed differences over extraction methods (**Table 2** **,** **Figure 4B**). For *A. judaica*, components showing large differences were artemisia ketone (53.6 and 40.3%, respectively), chrysanthenone (9.2 and 2.4%, respectively), artemisia alcohol (3.5 and 13.6%, respectively), yomogi alcohol (3.2 and 0.5%, respectively) and filifolide A (2 and 4.5%, respectively) (**Table 3** **,** **Figure 4C**).

**Table 1:** Volatile composition of *O. dayi* plant foliage (MTBE extract), in the soil in which *O. dayi* is growing (MTBE extract), and in the air surrounding the plant (SPME).

Compound	Plant foliage		Soil		Air	
	% oftotal	SD	% oftotal	SD	% oftotal	SD
1,8-Cineole	13.2	1.7	38.7	29.9	39.2	14.0
α-Terpineol	11.8	1.1	0.4	1.7	4.2	5.4
β-Pinene	8.9	0.8	0.6	2.4	7.4	2.5
Terpinen-4-ol	7.3	1.6	0.3	1.2	7.4	7.5
Linalyl acetate	7.0	1.7	n.d.		2.8	6.4
cis-Sabinene hydrate	6.7	1.8	0.5	1.4	0.9	1.6
trans-Sabinene hydrate	6.6	1.1	0.1	0.4	0.6	1.1
α-Pinene	5.6	0.5	9.1	1.2	7.7	3.2
γ-Terpinene	4.9	0.9	2.6	4.3	8.6	2.6
Sabinene	4.7	0.6	0.6	1.5	5.8	1.5
para-Cymene	3.9	1.3	2.7	10.5	7.0	3.3
Myrcene	3.8	0.5	0.3	1.3	2.4	2.1
α-Terpinyl propanate	3.8	1.4	n.d.		n.d.	
δ-Terpineol	2.7	0.2	0.1	0.3	0.4	0.6
Intermedeol	2.2	0.2	n.d.		n.d.	0.1
α-Thujene	2.1	0.3	0.6	1.1	2.9	0.8
(E)-Cryophyllene	1.8	0.2	0.1	0.5	0.3	0.5
Borneol	1.1	0.4	0.3	1.1	0.9	1.5
trans-Sabinenehydrate acetate	0.9	2.5	n.d.		n.d.	
cis-Methyl epi-jasmonate	0.4	0.1	n.d.		n.d.	
Limonene	0.4	0.1	42.6	37.4	1.1	0.5
Eugenol	0.3	0.1	n.d.		n.d.	
trans-Methyl jasmonate	0.2	0.0	n.d.		n.d.	
Vanillin	0.2	0.0	n.d.		n.d.	
Bornyl acetate	0.1	0.0	0.2	0.9	n.d.	
Neryl acetate	0.1	0.0	n.d.		0.1	0.2
α-Terpinyl acetate	0.1	0.0	n.d.		n.d.	

All the measurements were taken in the summer. n = 5–17, n.d. = not detected, 0 represents values less than 0.1.

**Table 2:** Volatile composition of *A. sieberi* plant foliage (MTBE extract), in the soil in which *A. sieberi* is growing (MTBE extract), and in the air surrounding the plant (SPME).

Compound	Plant foliage		Soil		Air	
	% oftotal	SD	% oftotal	SD	% oftotal	SD
1,8-Cineole	29.1	3.3	21.5	32.6	49.0	10.1
Camphor	28.5	3.1	7.4	13.4	4.8	4.2
Borneol	19.8	3.2	0.0	0.0	0.9	1.6
Germacrene D	14.4	3.5	0.0	0.1	0.0	0.0
Artemisia alcohol	13.0	2.2	0.0	0.0	0.6	0.8
trans-Thujone	10.4	1.5	45.7	42.0	13.0	15.2
para-Cymene	9.2	2.1	1.3	5.4	10.1	3.2
Camphene	9.1	1.1	4.8	6.6	3.4	1.8
Sabinene	2.7	0.6	0.3	0.9	6.1	2.8
α-Pinene	2.4	0.6	18.3	24.8	8.8	5.6
Pinocarvone	1.9	0.3	n.d.	0.0	0.2	0.4
Benzoic acid(methyl vanillate)	1.6	0.1	n.d.	0.0	n.d.	0.0
cis-Sabinene hydrate	1.5	0.3	n.d.	0.0	n.d.	0.1
trans-Sabinene hydrate	1.4	0.3	n.d.	0.0	n.d.	0.1
Myrtenol	1.3	0.3	n.d.	0.0	n.d.	0.0
Carvacrol	0.9	0.1	n.d.	0.0	n.d.	0.0
Jasmine ketolactone	0.8	0.1	n.d.	0.0	n.d.	0.0
(Z) Methyl jasmonate	0.7	0.1	n.d.	0.0	n.d.	0.0
α-Tujene	0.6	0.2	0.2	0.6	2.6	1.3
Eugenol	0.3	0.1	n.d.	0.0	n.d.	0.0
cis-Thujone	n.d.	0.0	0.4	0.8	0.3	0.8
Terpinen-4-ol	n.d.	0.0	n.d.	0.0	0.2	0.2

All measurements were taken in the summer. n = 5–17, n.d. = not detected, 0 represents values less than 0.1.

**Table 3:** Volatile composition of *A. judaica* plant foliage (MTBE extract), in the soil in which *A. judaica* is growing (MTBE extract), and in the air surrounding the plant (SPME).

Compound	Plant foliage		Soil		Atmosphere	
	% oftotal	SD	% oftotal	SD	% oftotal	SD
Artemisia ketone	33.3	0.6	67.2	31.1	81.3	4.2
(E) Ethyl cinnamate	18.3	0.5	5.3	15.1	0.2	0.6
Davanone	9.6	1.7	n.d.		n.d.	
Artemisia alcohol	8.6	1.1	0.6	1.7	0.8	1.2
Filifolide A	8.5	1.3	12.7	9.4	0.7	2.4
(Z) Ethyl cinnamate	6.4	0.4	0.5	1.1	0.2	0.7
Piperitone	5.7	0.4	2.7	7.8	0.1	0.4
β-Davanone-2-ol	2.7	0.1	n.d.		n.d.	
Chrysanthenone	1.6	0.2	0.2	0.7	2.6	2.3
nor-Davanone	1.0	0.1	0.1	0.3	n.d.	
Borneol	1.0	0.1	0.1	0.2	0.1	0.2
Yomogi alcohol	0.8	0.1	0.3	0.7	1.0	0.7
Camphor	0.7	0.1	8.9	14.4	2.1	1.8
Methyl vanillate	0.6	0.2	n.d.		n.d.	
Sabinene	0.5	0.1	1.4	5.4	10.9	5.0
Jasmine ketolactone	0.2	0.0	n.d.		n.d.	
Methyl epi-jasmonate	0.2	0.0	n.d.		n.d.	
Methyl jasmonate	0.1	0.0	n.d.		n.d.	

All measurements were taken in the summer. n = 5–17, n.d. = not detected, 0 represents values less than 0.1.

#### Changes in volatile content with seasons

**Figure 5:**
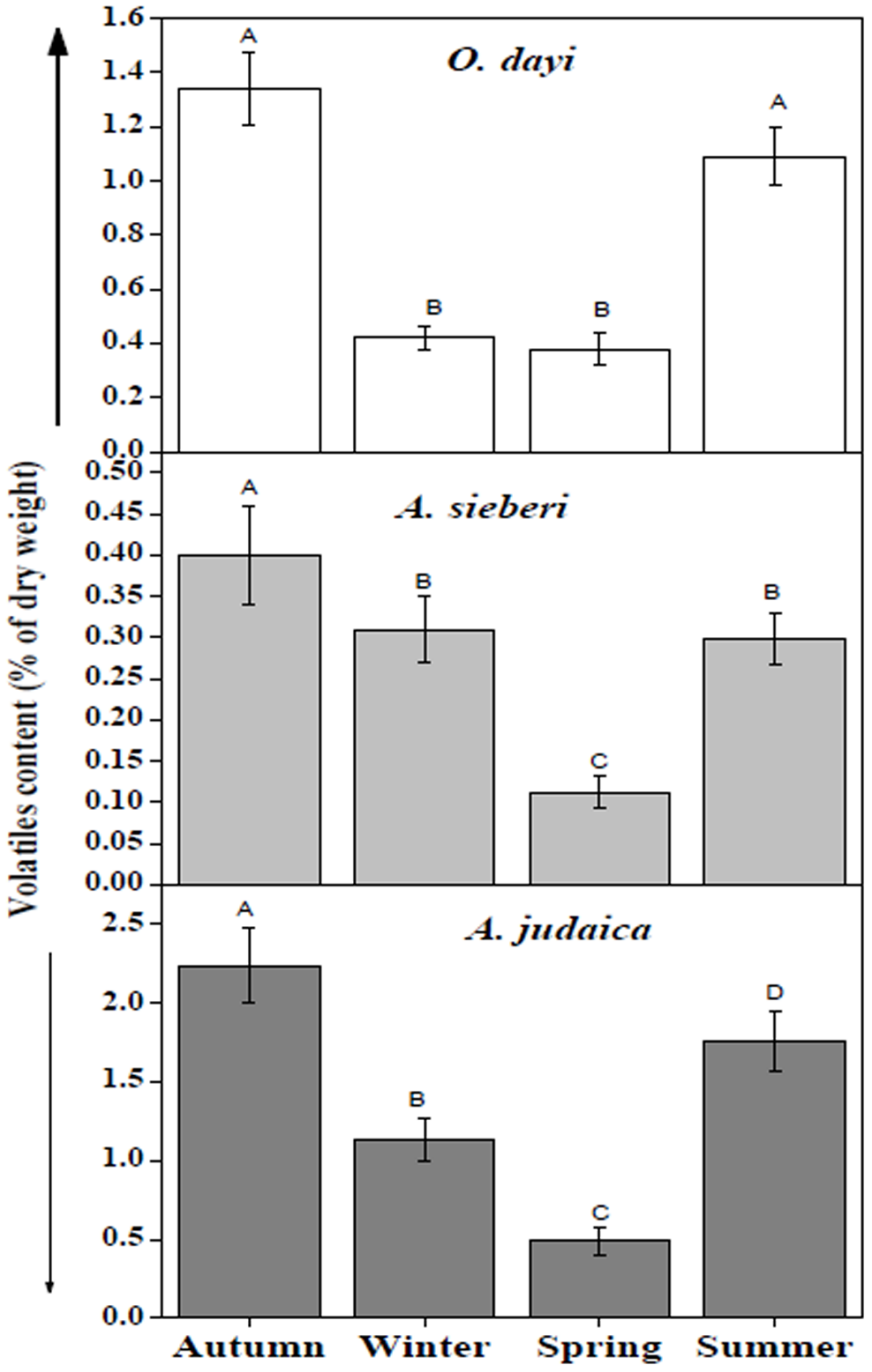
Change in volatile contents with the seasons in *O. dayi* (upper panel), *A. sieberi* (middle panel) and *A. judaica* (lower panel), n = 18. Error bars represent standard deviation. Different uppercase letters represent significant differences between seasons (*P*<0.05).

For *O. dayi*, there were significant changes (*P*<0.05) in the relative percentage (of plant dry weight) of total compounds as measured following extraction in organic solvent (**Figure 5** **upper panel**). The highest concentration was measured in the autumn–1.3%, and the lowest in the spring–0.4%. In *A. sieberi*, the highest concentration of total compounds was measured in the autumn–0.4%, and the lowest in the spring–0.1% (**Figure 5** **middle panel**). For *A. judaica*, the highest concentration was measured in the autumn–2.3%, and the lowest in the spring–0.4% (**Figure 5** **lower panel**).

#### Changes in volatile composition with season

A comparison of volatile composition in the different seasons (**Tables S1–S3**) showed that some components change dramatically with the seasons in relative percentage of total volatiles. For example in *O. dayi,* linalyl acetate (2.5% in the spring, 10.3% in the autumn), cis-sabinene hydrate (2.5% in the spring, 10.3% in the autumn), α-terpineol (6.6% in the spring, 11.8% in the summer), sabinene (4.7% in the summer, 8.1% in the spring) and trans-sabinene hydrate (1.8% in the autumn, 6.6% in the summer) showed significant differences among seasons (**Table S1**). In *A. sieberi*, 1,8-cineole (7.9% in the winter, 29.1% in the summer), camphor (8% in the spring, 28.5% in the summer), artemisia alcohol (5.8% in the winter, 13% in the summer), trans-thujone (1.6% in the spring, 19.7% in the winter), borneol (2.5% in the winter, 19.8% in the summer), germacrene D (3% in the winter, 14.4% in the summer) and para-cymene (0.5% in the spring, 9.2% in the summer) showed significant differences among the seasons (**Table S2**). In *A. judaica*, ethyl cinnamate (11.4% in the spring, 18.3% in the summer), filifolide A (4.5% in the spring, 8.5% in the summer) and piperitone (2.2% in the spring, 5.7% in the summer) showed seasonal differences in composition (**Table S3**).

The summer season was characterized with water deficit conditions when compared with the winter season (**Figure 1**). We observed that the relative percentage of some of the volatiles was significantly increased during summer season over the winter. For instance of the following volatiles increased in summer season over the winter period in *O*. *dayi*, Linalyl acetate (32%), Terpinen-4-ol (40%), γ-Terpinene (35%), trans-sabinene hydrate (73%) and Trans-Sabinene hydrate acetate (44%); in *A. sieberi*, 1,8-cineole (73%), camphor (69%), artemisia alcohol (66%), borneol (87%), germacrene D (79%), camphene (67%), para-cymene (78%), trans-Sabinene hydrate (71%), pinocarvone (63%), jasmine ketolactone (62%) and α-Tujene (50%); in *A. judaica*, ethyl cinnamate (38% increase over spring), borneol (40%) and piperitone (33%). Enhanced volatile content during summer season may be responsible for imparting drought tolerance of the plants. This selective enhancement in metabolites under water deficit conditions (summer season) may be related with improved plant height and stem diameter in *O*. *dayi* and *A*. *sieberi* (**Figure 2** **and** **3**).

### Allelopathic Effects and Survival of Neighboring Plants

#### In-vitro allelopathic effects and seed germination

**Figure 6:**
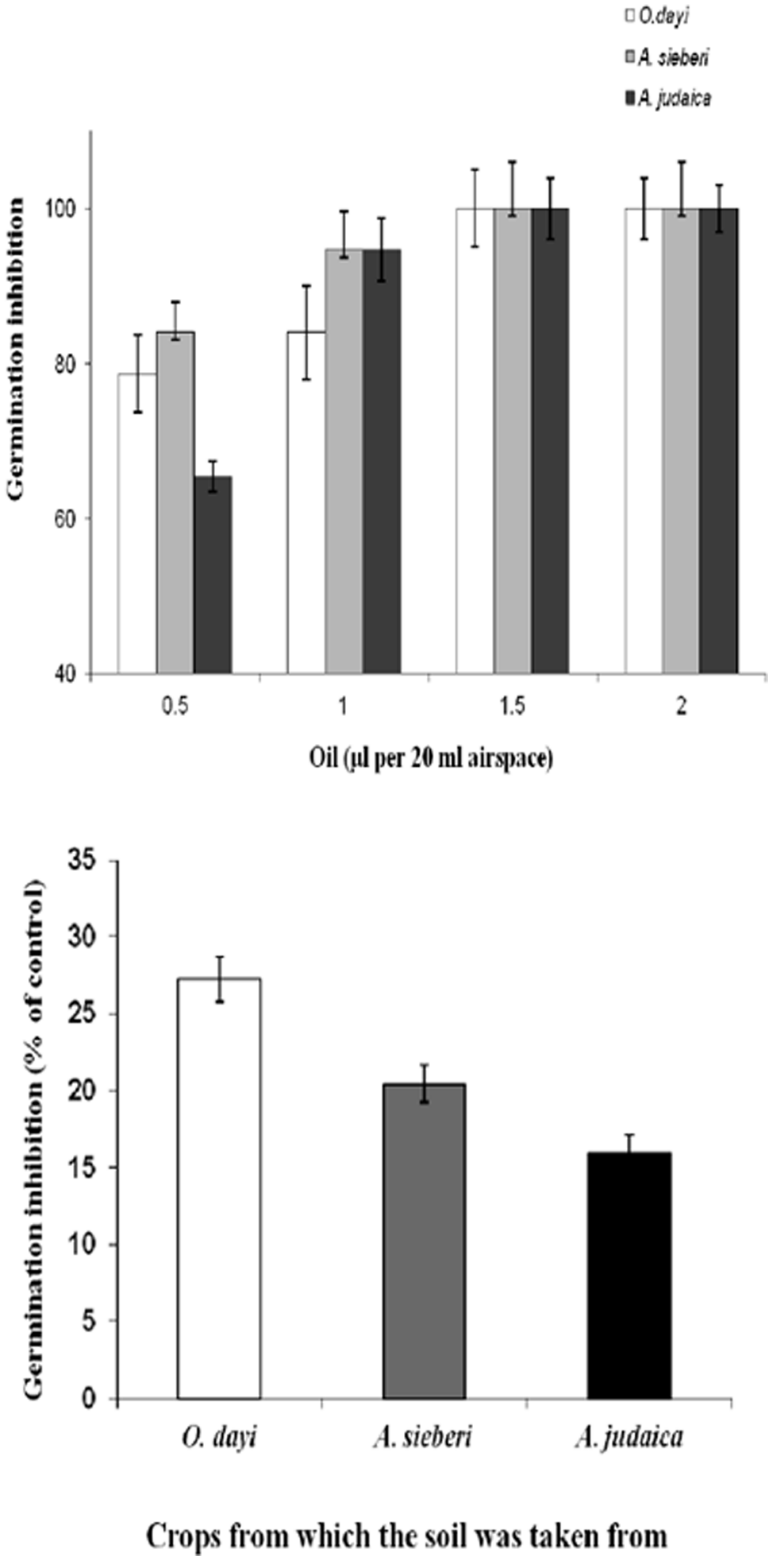
Allelopathic effects of essential oils of *O. dayi*, *A. sieberi* and *A. judaica*. Inhibitory effects of essential oils from the three species at four different concentrations on wheat germination *in vitro* (upper panel) and of soil samples taken from under the canopies of the three species (lower panel), n = 5. Error bars represent standard error, n.s. = not significant.

In the two experiments carried out under control conditions, germination of wheat seeds was inhibited (**Figure 6** **upper panel**). In the essential oil inhibition experiment, although there were insignificant differences (P>0.05) with low concentrations of essential oil (0.5 µl per 20 ml airspace), *A. sieberi* showed the highest inhibition (78.8%) and *A. judaica* the lowest (65.3%). However, high concentration (1.5 µl per 20 ml airspace) of oil from all the three species caused 100% inhibition (**Figure 6** **upper panel**).

In the soil inhibition experiment (**Figure 6** **lower panel**), although there were insignificant differences (*P*>0.05), soil taken from plots in which *O. dayi* was growing caused the highest inhibition (27.3%) and soil in which *A. judaica* was growing caused the lowest inhibition (15.9%) of wheat seed germination (**Figure 6** **lower panel**).

#### In-vivo allelopathic effects in the field

**Figure 7:**
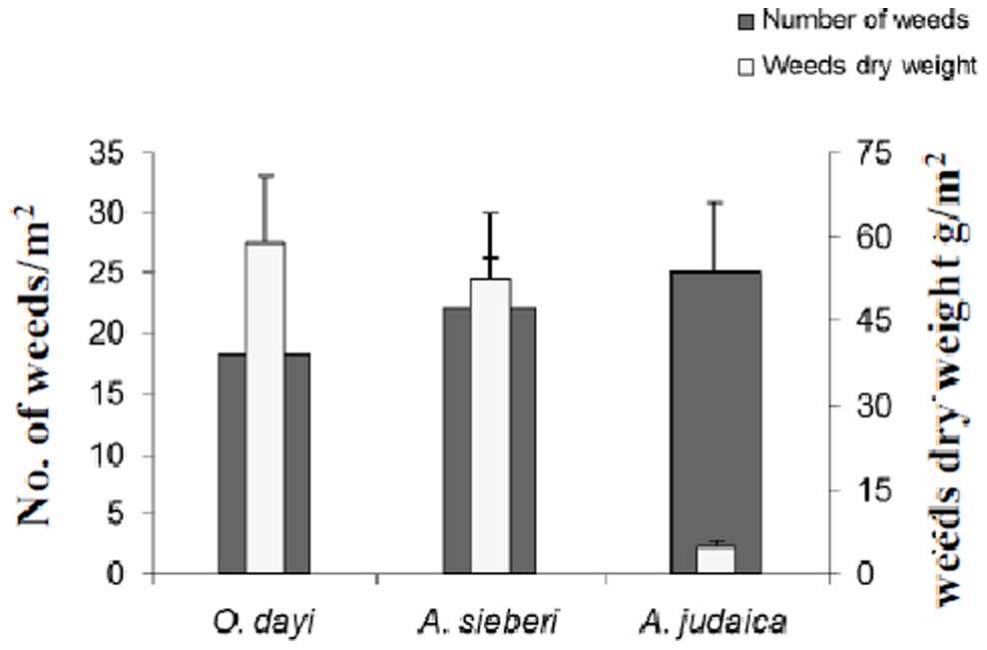
Number of weeds and weed biomass in the field. Relationship between number of weeds and their dry weight in the three species’ plots. All measurements were taken in the autumn, n = 9. Error bars represent standard error.

**Figure 8:**
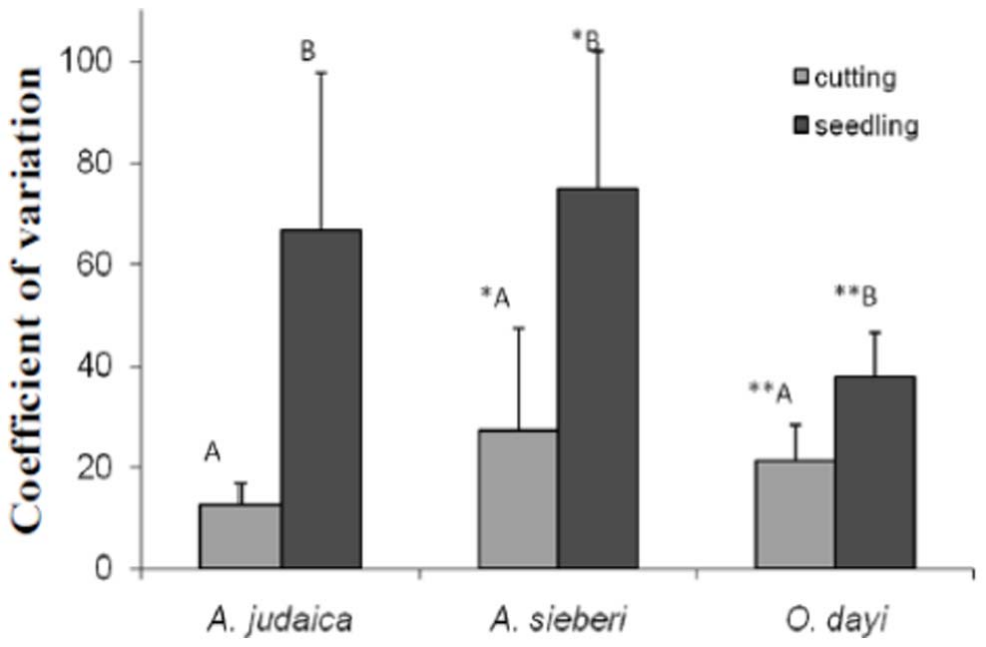
Representatives of the different weed families in the different species’ plots. Relative % of weeds from major botanical families out of total number of weeds in the different species’ plots. All measurements were taken in the autumn, n = 9. Error bars represent standard error.

For the three species under study, different ratios were found between the number of weeds and their biomass (dry weight) (**Figure 7**). In *O. dayi* plots, a low number of weeds and high weight gave the ratio of 0.038. In contrast, in *A. judaica* plots, a high number of weeds and low weight gave the ratio 0.0025 (**Figure 7**). For *A. sieberi*, the weed number-to-weight ratio was 0.032 (**Figure 7**). Furthermore, a specific effect of the allelopathic response was observed when the weeds growing in the three species’ plots were classified into botanical families (**Figure 8**). With *A. sieberi* and *A. judaica*, there were higher differences in the number of weeds belonging to the different botanical families than with *O. dayi* (**Figure 8**).

#### Effects of allelopathy on volatiles present in the soil and air

Soil samples extracted in the organic solvent and air samples were chemically analyzed for volatile identification. Although both air and soil detection methods under field conditions were not sufficiently accurate, and identified volatiles were mixed with those from the surrounding environment, a high correlation was found between major volatile compounds in the plant extract and those found in the soil and air samples. In *O. dayi*, the major component in the plant–1,8-cineole, was found in very high concentrations in both the air and soil. α-Terpineol, β-pinene and terpinen-4-ol were found at high concentrations in the plant and in the air (**Table 1**). In *A. sieberi*, 1,8-cineole, also the major component in the plant, was again found at very high concentrations in both air and soil. Camphor and para-cymene were found at intermediate and high concentrations in the plant, soil and air (**Table 2**). In *A. judaica*, the major component in the plant–artemisia ketone–was found at very high concentrations in both in air and soil. Filifolide A was found at high concentrations in the plant extract and in the soil (**Table 3**).

### Comparing Seedlings and Cuttings as a Source of Variance

#### Volatiles’ coefficients of variation among species

**Figure 9:**
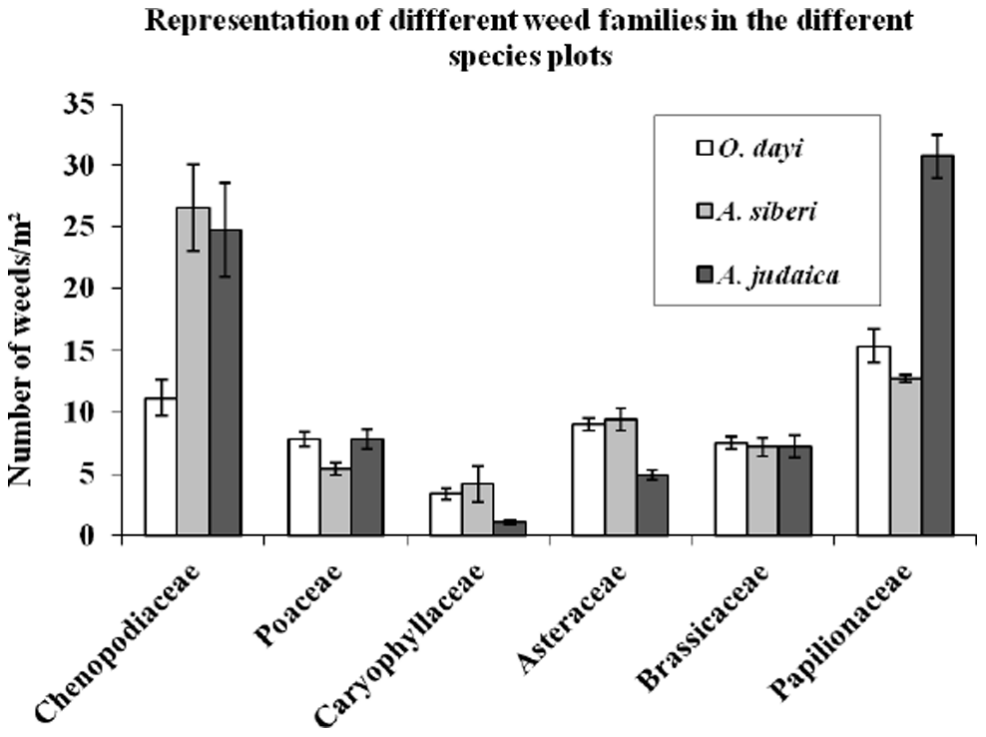
Coefficient of variation of volatiles (volatile composition) in cuttings and (volatile composition) seedlings. Average volatile composition coefficient of variation in the three species, comparing seedlings (dark gray) and cuttings (light gray) extracted with organic solvents. All measurements were taken in the autumn, n = 17–18. Error bars represent standard error.

High coefficients of variation in the seedling populations of *O. dayi*, *A. sieberi* and *A. judaica* were found relative to the populations of cuttings (**Figure 9**). In addition, the highest values in the seedling population were found for *A. sieberi* (74.7) and the lowest for *O. dayi* (37.8).

#### Comparative analysis of volatile composition among species

Comparison of the relative percentages of the volatile components in the three species showed that in each species, there were several components with high differences between seedlings and cuttings. The highest differences in *A. sieberi* (data not shown) were found for the compounds trans-thujone (36.5% in seedlings, 5.5% in cuttings), 1,8-cineole (18% in seedlings, 12% in cuttings), sabinene (7.2% in seedlings, 0.5% in cuttings), para-cymene (6.1% in seedlings, 2.9% in cuttings), cis-sabinene hydrate acetate (5.1% in seedlings, 0% in cuttings) and camphor (0.5% in seedlings, 11.7% in cuttings). In *O. dayi*, the highest differences were found for α-terpineol (6.1% in seedlings, 10.6% in cuttings), linalyl acetate (5.5% in seedlings, 9.9% in cuttings) and cis-sabinene hydrate (3.8% in seedlings, 10.4% in cuttings). In *A. judaica*, the highest differences were found for filifolide A (11.8% in seedlings, 7.7% in cuttings), davanone (2.7% in seedlings, 11.5% in cuttings) and camphor (18% in seedlings, 12% in cuttings) (data not shown).

### Comparative PCA Analyses

**Figure 10:**
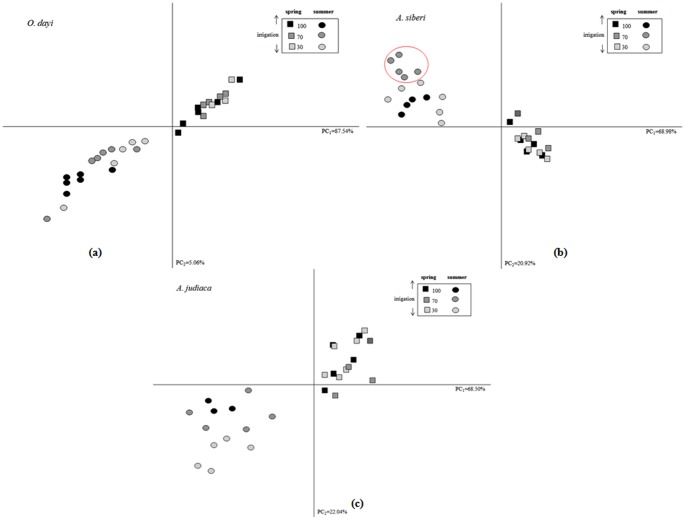
Principal component analysis (PCA) of *O. dayi* (A), *A. sieberi* (B) and (C) *A. judaica* subjected to the four season regimes.

In *A. sieberi*, plants in the spring and summer seasons showed no significant changes in PCA (**Figure 10A** **, Table S4B**). The distribution pattern of metabolites showed visible separation on the PCA plot for *A. judaica* in the summer, whereas no such separation was noted for the spring season (**Figure 10B** **, Table S4A**). No clear separation was observed for *O. dayi* plants in either summer or spring (**Figure 10C** **, Table S4C**) under low water conditions.

## Discussion

Allelopathy is a well-studied phenomenon in which chemicals released by one plant may have detrimental or inhibitory effects on the germination, seedling establishment or growth pattern of neighboring plants [8]–[9], [18]–[19]. In fact, allelochemicals produced by plants serve many purposes aside from being active in allelopathy, such as avoidance of herbivory and reducing competition for light, water and other micro- and macronutrients [37]. Desert plants have been known to survive drought periods more successfully than mesophytes and other terrestrial plant groups [9], [18]–[19]. However, the role of secondary metabolites in the survival of desert plants in conjunction with allelopathy has never been explored, and offers a new research vista. The present study was therefore performed to elucidate the role of alleochemicals in survival under arid or semiarid desert conditions and in allelopathy, with a major focus on the botanical use of plant extracts for weed control. Our findings are the first to report a role for allelopathy in the successful survival of desert plants with agronomical use of plant extracts of the species under study to handle weed problems.

### Allelopathy and Water Stress

The plants under study were grown under natural conditions with no surplus water provided, replicating their natural habitat niche. Significant amounts of soil water content were noted in winter vs. summer soils (**Figure 1**). These seasonal changes in soil water content can be linked to less evapotranspiration in the winter than in the summer [2]. Differences were expected in the composition and content of metabolites identified in the three species facing the four different seasonal regimes. Seasonal variations were found to have a significant impact on composition and content of specific metabolites that are held responsible for allelopathy (**Tables S1–S3**). The concentrations of these metabolites differed significantly across the three analyzed samples, i.e. in the plant foliage, soil and air (discussed further on) (**Tables 1** **–** **3** **,** **Figure 4**). Plant growth (height and stem diameter) was differentially affected across the three species in the four seasons, with most of the growth changes in all three studied species observed in the autumn and winter (**Figures 2** **,** **3**). Significant differences in leaf RWC were noted for *O. dayi*, *A. sieberi* and *A. judaica* across the different seasons (**Figure S1**), which may be associated with changes in water level, humidity and temperature induced seasonal changes [38]. These seasonal changes were also found to significantly affect the osmolarities of all the three species, albeit differentially (**Figure S2**). Interseasonal changes in photochemical activity were noted in all species studied. These subtle changes could be linked to differences in light intensity in the different seasons [39].

### Influence of Allelopathic Abilities on Weed Populations

All three species showed allelopathic abilities in the laboratory experiments with respect to germination inhibition by essential oils (**Figure 6** **upper panel**) and by soils (**Figure 6** **lower panel**). This enabled us to conclude that allelopathic inhibition can be caused by volatiles in the air (as reflected by the essential oil germination inhibition experiment), inhibitory components in the soil (as seen in the soil germination inhibition experiment) or a combination of air- and soilborne allelopathic factors. The latter case may be supported by the presence of compounds in the plant’s atmosphere in both the field and the soil in which the plants grew (**Tables** **1** **–** **3** **,** **Figure 6**).

The allelopathic tendencies of the genus *Artemisia* have been well demonstrated. The main allelopathic compounds in this genus include artemisinin [40], methyl jasmonate [41], camphor and cineole epimers [42]. Allelopathic effects of *A. judaica* have been demonstrated with its dry powder, showing inhibition of lettuce germination and growth [43]. The essential oil of *A. judaica* has also been shown to have high germination-inhibition abilities in comparison to other species [33]. Germination inhibition of *Helianthemum squamatum* seeds by *A. sieberi* shoot extract was reported by [27].

An extensive literature survey showed no previous information on the allelopathic abilities of *O. dayi*, whereas other species of this genus, such as *O. syriacum, O. vulgare,* and *O. majorana*, are known to possess low germination-inhibition abilities relative to other species [33]. In the present study, the highest levels of germination inhibition at lowest concentration applied (0.5 µl) were obtained with the essential oil from *O. dayi* and *A*. *sieberi* **(** **Figure 6** **upper panel)** and with *O. dayi* in soil induced germination inhibition (**Figure 6** **lower panel)**. Among three species under study *A. judaica* showed lowest weeds dry weight, while lowest number of weeds was recorded for *O. dayi* (**Figure 7**).

Specificity of allelopathic effects was noted in *A. judaica* plots, which showed the largest differences between the weed varieties growing in their plots (**Figure 8**). The highest number of weeds affected by *A. judaica* belonged to the *Papilonaceae*, whereas in *A. sieberi* plots, the highest number of affected weeds belonged to the *Chenopodiaceae* (**Figure 8**). In *O. dayi* plots, the changes were minor, with the dominant weeds belonging to the Papilonaceae (**Figure 8**). Previous allelopathic studies have shown that different plant species often respond differently to allelochemicals, presumably due to variations among evolutionary histories. This study’s findings should be helpful in creating specific herbicides for weeds by using different quantities of oils from the different species according to various needs.

### Metabolites and Allelopathy in *O. Dayi*, *A. Sieberi* and *A. Judaica*

Among the metabolites identified in *O. dayi*, 1,8-cyneole was present at the highest concentration in solvent, soil and air samples, indicating its abundance in and around the plant (**Table 1** **,** **Figure 4**). On the other hand, compounds like α-terpineol, α-pinene, terpinen-4-ol, linalyl acetate and cis-sabinene hydrate were present at high concentrations in the solvent and the air around the foliage, but were absent or at low concentrations in the soil samples. Interestingly, limonene content in the soil sample was 100-fold than that in the solvent sample, and this compound was entirely absent in the air sample (**Table 1** **,** **Figure 4**). The high concentration of limonene in the soil suggests a crucial role in allelopathic effects such as inhibition of seed germination and seedling establishment.

The concentration of compounds released by *A. sieberi* and its allelopathic effects were also correlated. 1,8-Cineole and camphor were present in great abundance in all three samples analyzed, i.e. plant foliage, soil and air (**Table 2** **,** **Figure 4**). Trans-thujone and α-pinene were present at higher concentrations in the soil and air samples than in the foliage sample, indicating their significant role in allelopathic effects. The concentration of limonene found in the soil sample was much higher than in the foliage or soil samples, thereby indicating its role in producing allelopathic effects on plants growing in *A. sieberi*’s vicinity. In contrast, α-tujene was present in high abundance in air samples relative to soil and foliage samples. A high concentration of α-tujene might be responsible for this plant’s airborne allelopathic effects (**Table 2** **,** **Figure 4**).

The allelopathic effects of *A. judaica* were regulated by a large number of natural compounds released by the plants, some of them residing in the plant tissue–leaves, stem and roots–and others passed into the soil and air to regulate allelopathic reactions occurring there. Among the metabolites identified in *A. judaica*, artemisia ketone and ethyl cinnamate were present in the foliage, soil and air samples (**Table 3** **,** **Figure 4**); however, concentrations of these compounds were much higher in the soil and air samples than in the plant sample. The concentration of filifolide A was much higher in the soil sample than in the plant or air samples, suggesting this metabolite’s greater contribution to soil borne allelopathic effects (**Table 3** **,** **Figure 4**). Concentrations of chrysanthenone and sabiene were much higher in air samples than in soil and plant samples indicating their important role in allelopathic regulation of neighboring plants through inhibition of seed germination and seedling establishment.

### Why do Desert Plants Thrive under Arid and Semiarid Conditions?

Extensive GC-MS analyses of *O. dayi*, *A. sieberi* and *A. judaica* under the four seasonal regimes which differed in soil water level revealed some interesting facts. Although insignificant differences were found in the relative content of most of the metabolites identified under low soil water (summer) and high soil water (winter) regimes (**Tables S1–S3**). Only few metabolites showed significant increases in their relative content in the summer season (water deficit conditions) when compared to winter season (high soil water). This selective increase in the relative metabolite content may be associated with enabling plants under study to thrive efficiently under water deficit conditions. For instance elevated levels of linalyl acetate, terpinen-4-ol, γ-terpinene, trans-sabinene hydrate and trans-sabinene hydrate acetate in *O*. *dayi*; 1,8-cineole, camphor, artemisia alcohol, borneol, germacrene D, camphene, para-cymene, trans-Sabinene hydrate, pinocarvone, jasmine ketolactone and α-Tujene in *A. sieberi*; ethyl cinnamate, borneol and piperitone in *A. judaica* were observed. Enhanced volatile content of only few metabolites among an array of metabolites suggested their selective induction during summer season over winter period. Such that five out of twenty seven in *O*. *dayi*; eleven out of twenty four in *A*. *sieberi* and three out of seventeen metabolites in *A*. *judaica* were shown to be significantly enhanced under water deficit period during summer season, and this selective metabolomic shift may be responsible for improved plant growth and physiological attributes such as Fv/Fm which in turn may be involved in imparting drought resistance of plants under study (**Tables S1–S3,** **Figure 5**).

This resistance to a major shift in metabolome content and composition with changing seasons might be a crucial factor in the successful survival of these plant species under harsh and changing environments [44]. PCA led us to propose that *O. dayi* is more resistant to seasonal variations than *A. sieberi* or *A. judaica* (**Figure 10**). Metabolite production also did not vary significantly in the three species studied, indicating their potential to thrive under water stress without jeopardizing their metabolic regulation drastically. Furthermore maintenance of a threshold level of specific metabolites might be a key to the successful survival of these plant species under subtle changes in soil water status. Moreover, during the course of their evolution, these plant species have learned to maintain the homeostatic balance of natural compounds, enabling them to survive longer periods of drought and harsh environments [44].

## Conclusion

The physiological conjunction of allelochemicals in desert plants and their role in plant survival during periods of low water state is shown for the first time. Our findings showed that water deficit condition is the driving force for metabolome shift in plants growing in arid or semi-arid soil conditions. Selective induction of few metabolites and keeping significant part of the metabolome unaffected by water deficit condition may be a key strategy for water stress management in the desert plants. Current study also suggests that homeostasis in the endogenous levels of natural compounds plays a crucial role in these species’ ability to thrive during periods of water stress. Findings also showed the participation of natural compounds in the allelopathic behavior of *O. dayi*, *A. sieberi* and *A. judaica*, which may serve as a basis for the generation of species-targeted herbicides. A detailed molecular insight of desert plants under water stress condition will be a great future endeavor.

## Supporting Information

Figure S1 **Leaf relative water content (RWC) in the different seasons in** ***O. dayi*** **(white bars),** ***A. sieberi*** **(light gray bars) and** ***A. judaica*** **(dark gray bars).** n = 5–6 and error bars represent standard deviation. Different uppercase letters represent differences within the seasons.
(TIF)

Figure S2 **Osmolarity (measured by pressure osmometer) in the different seasons in** ***O. dayi*** **(white bars),** ***A. sieberi*** **(light gray bars) and** ***A. judaica*** **(dark gray bars).** n = 5–6 and error bars represent standard deviation. Different uppercase letters represent differences within the seasons.
(TIF)

Figure S3 **Photochemical activity: changes in effective quantum yield (Fv/Fm) in the different growing seasons in** ***O. dayi*** **(white bars) and** ***A. judaica*** **(dark gray bars).** n = 6. error bars represent standard deviation. Different uppercase letters represent differences within seasons.
(TIF)

Table S1 **Changes in the composition of volatiles in organic-solvent extract of** ***O. dayi*** **with the seasons–only 27 major components are shown.** n = 18, n.d. = not detected, 0 represents values less than 0.1.
(DOC)

Table S2 **Changes in the composition of volatiles in organic-solvent extract of** ***A. sieberi*** **with the seasons–only 24 major components are shown.** n = 18, n.d. = not detected, 0 represents values less than 0.1.
(DOC)

Table S3 **Changes in the composition of volatiles in organic-solvent extract of** ***A. judaica*** **with the seasons–only 17 major components are shown.** n = 18, n.d. = not detected, 0 represents values less than 0.1.
(DOC)

Table S4 **Eigenvector values (in descending order) of metabolites were calculated by PCA algorithm for 1st and 2nd components of targeted samples in** ***A. judaica*** **(A),** ***A. sieberi*** **(B) and** ***O. dayi*** **(C).**
(DOC)
