# Supplementary figures and images for: Physiological Conjunction of Allelochemicals and Desert Plants
Source: PLoS One. 2013 Dec 10;8(12):e81580. doi: 10.1371/journal.pone.0081580 (PMC3858270; doi:10.1371/journal.pone.0081580)

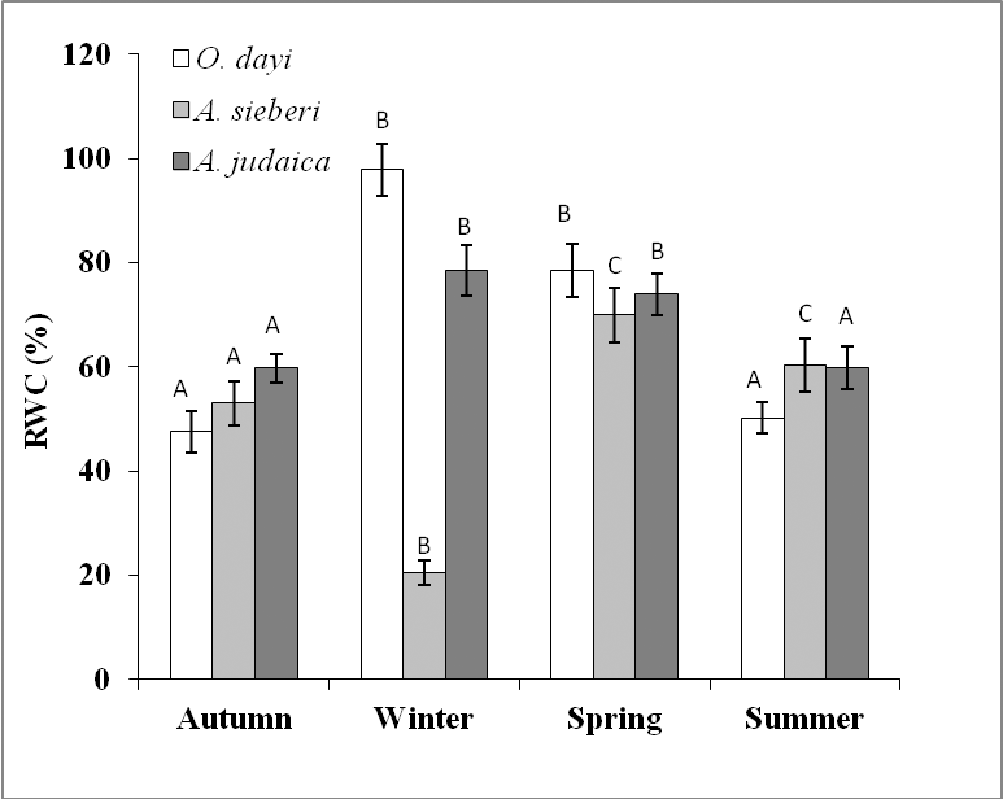

Supplement: Figure S1 — Leaf relative water content (RWC) in the different seasons in O. dayi (white bars), A. sieberi (light gray bars) and A. judaica (dark gray bars). n = 5–6 and error bars represent standard deviation. Different uppercase letters represent differences within the seasons. (TIF) [file pone.0081580.s001.tif]

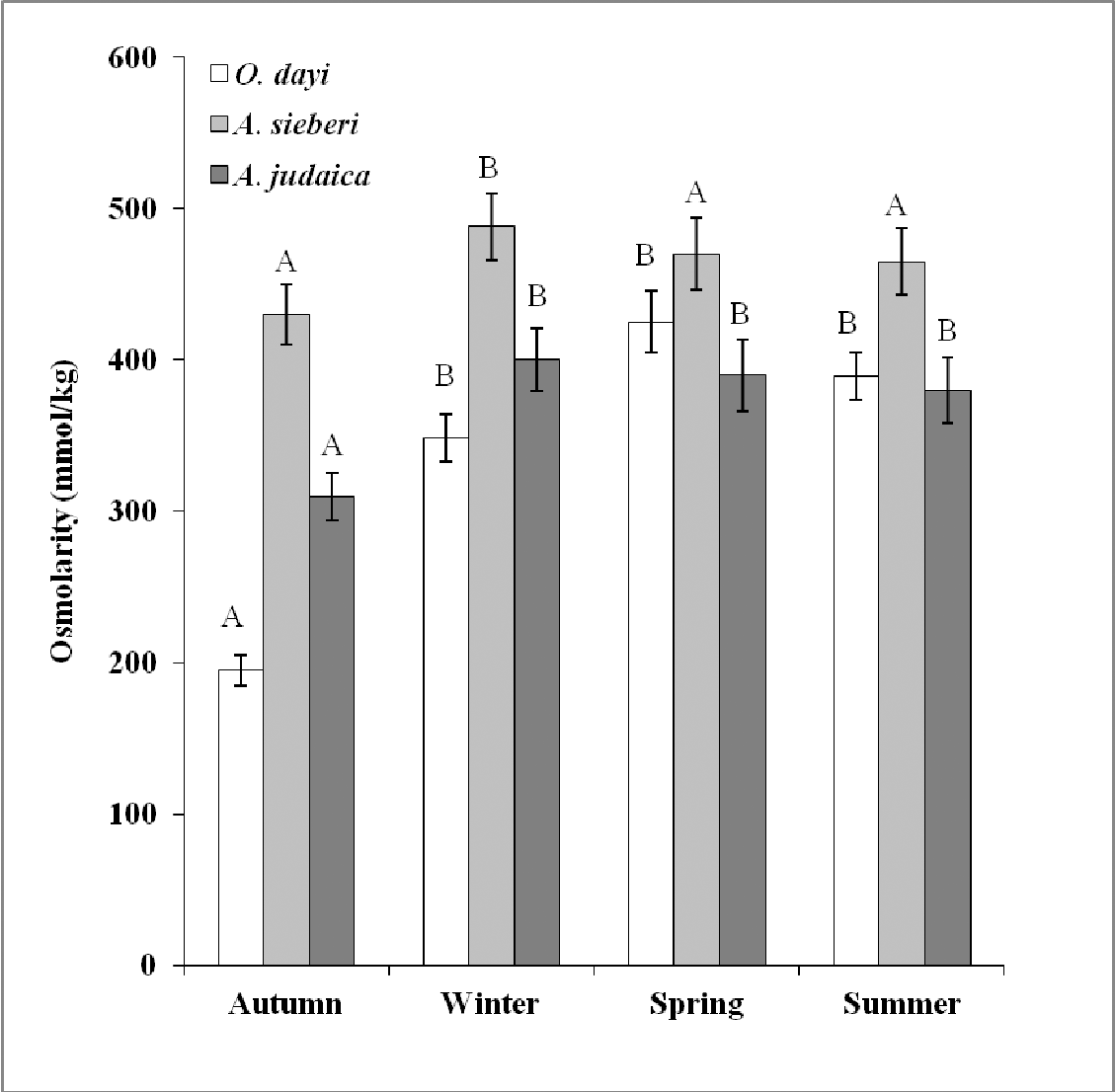

Supplement: Figure S2 — Osmolarity (measured by pressure osmometer) in the different seasons in O. dayi (white bars), A. sieberi (light gray bars) and A. judaica (dark gray bars). n = 5–6 and error bars represent standard deviation. Different uppercase letters represent differences within the seasons. (TIF) [file pone.0081580.s002.tif]

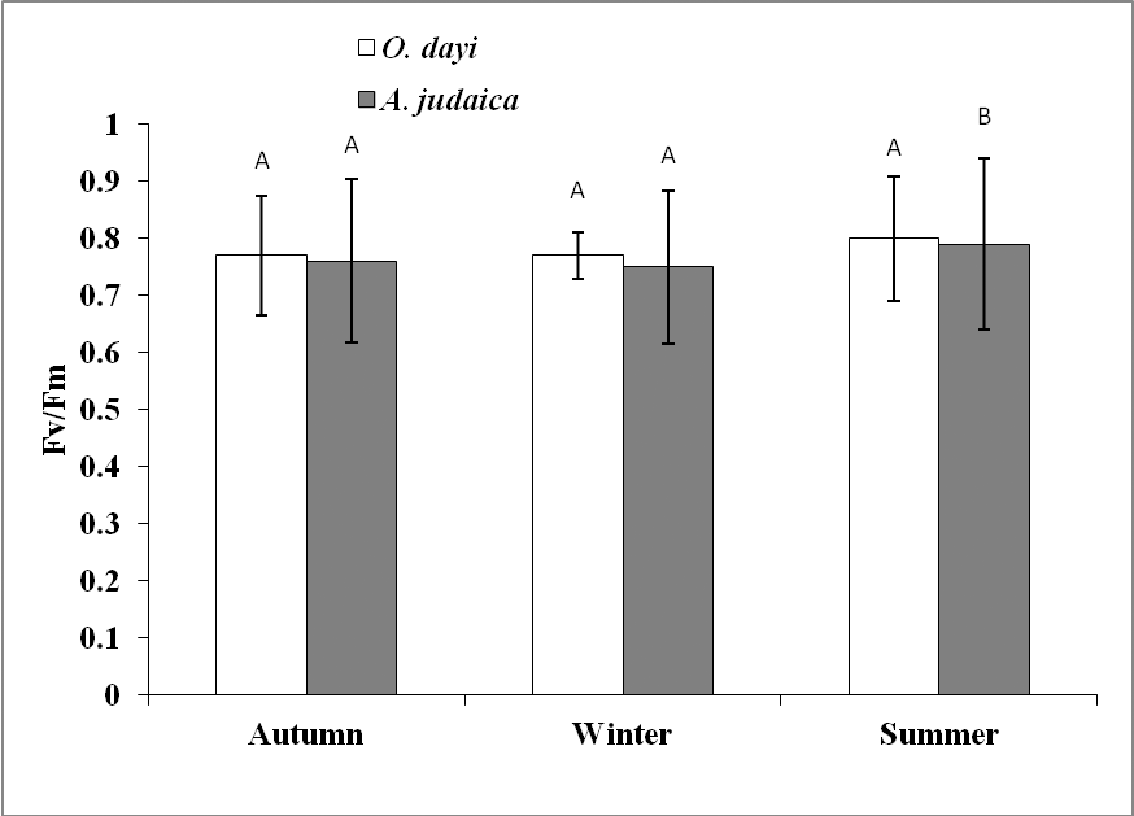

Supplement: Figure S3 — Photochemical activity: changes in effective quantum yield (Fv/Fm) in the different growing seasons in O. dayi (white bars) and A. judaica (dark gray bars). n = 6. error bars represent standard deviation. Different uppercase letters represent differences within seasons. (TIF) [file pone.0081580.s003.tif]
