# Supplementary material for: Physiological Conjunction of Allelochemicals and Desert Plants
Source: PLoS One. 2013 Dec 10;8(12):e81580. doi: 10.1371/journal.pone.0081580 (PMC3858270; doi:10.1371/journal.pone.0081580)
Supplement: Table S1 — Changes in the composition of volatiles in organic-solvent extract of O. dayi with the seasons–only 27 major components are shown. n = 18, n.d. = not detected, 0 represents values less than 0.1. (DOC) [file pone.0081580.s004.doc]

**Table S1.** Changes in the composition of volatiles in organic-solvent extract of *O. dayi* with the seasons—only 27 major components are shown. n = 18, n.d. = not detected, 0 represents values less than 0.1.

| **Compound** | **Autumn** | | **Winter** | | **Spring** | | **Summer** | |
| --- | --- | --- | --- | --- | --- | --- | --- | --- |
|  | **% of total** | **SD** | **% of total** | **SD** | **% of total** | **SD** | **% of total** | **SD** |
| 1,8-Cineole | 13.5 | 2.8 | 12.7 | 1.7 | 12.3 | 0.6 | 13.3 | 1.7 |
| Linalyl acetate | 10.3 | 2.3 | 4.8 | 2.2 | 2.5 | 0.5 | 7.1 | 1.7 |
| cis-Sabinene hydrate | 10.1 | 3.4 | 10.9 | 4.3 | 13.1 | 0.4 | 6.7 | 1.8 |
| α-Terpineol | 9.7 | 1.5 | 9.7 | 1.8 | 6.6 | 0.6 | 11.8 | 1.1 |
| β-Pinene | 9.6 | 1.6 | 8.4 | 0.9 | 10.2 | 0.8 | 8.9 | 0.8 |
| Sabinene | 7.2 | 0.9 | 7.3 | 0.9 | 8.1 | 1.3 | 4.7 | 0.6 |
| α-Pinene | 6.2 | 0.5 | 5.9 | 0.9 | 7.2 | 0.1 | 5.6 | 0.5 |
| Terpinen-4-ol | 4.0 | 1.1 | 4.4 | 1.0 | 4.5 | 1.0 | 7.3 | 1.6 |
| α-Terpinyl propanoate | 4.0 | 2.0 | 4.6 | 2.2 | 5.1 | 0.4 | 3.8 | 1.4 |
| Myrcene | 3.7 | 0.6 | 3.6 | 0.4 | 3.5 | 1.5 | 3.8 | 0.5 |
| δ-Terpineol | 3.1 | 0.4 | 2.1 | 0.3 | 1.9 | 0.5 | 2.7 | 0.2 |
| γ-Terpinene | 3.0 | 1.2 | 3.2 | 0.5 | 3.6 | 0.2 | 4.9 | 0.9 |
| Intermedeol | 2.6 | 0.2 | 2.5 | 0.5 | 2.3 | 0.9 | 2.2 | 0.2 |
| α-Thujene | 2.5 | 0.7 | 3.8 | 1.0 | 3.8 | 1.1 | 2.1 | 0.4 |
| (E)-Caryophyllene | 2.1 | 0.3 | 2.5 | 0.3 | 3.0 | 1.4 | 1.8 | 0.2 |
| para-Cymene | 1.9 | 0.6 | 2.3 | 0.8 | 3.5 | 1.2 | 3.9 | 1.3 |
| trans-Sabinene hydrate | 1.8 | 0.5 | 1.9 | 1.0 | 3.2 | 0.6 | 6.6 | 1.1 |
| Borneol | 0.7 | 0.5 | 2.1 | 0.9 | 2.6 | 0.5 | 1.1 | 0.5 |
| Limonene | 0.5 | 0.2 | 0.7 | 0.2 | 0.3 | 0.0 | 0.4 | 0.1 |
| Eugenol | 0.3 | 0.1 | 0.4 | 0.2 | 0.4 | 0.0 | 0.3 | 0.1 |
| cis-Methyl epi-jasmonate | 0.3 | 0.1 | 0.2 | 0.1 | 0.2 | 0.1 | 0.4 | 0.1 |
| trans-Methyl jasmonate | 0.2 | 0.1 | 0.2 | 0.1 | 0.1 | 0.0 | 0.2 | 0.0 |
| Neryl acetate | 0.2 | 0.1 | 0.1 | 0 | n.d |  | 0.1 | 0.0 |
| Bornyl acetate | 0.1 | 0.1 | 0.4 | 0.4 | 0.2 | 0.2 | 0.1 | 0.0 |
| α-Terpinyl acetate | 0.1 | 0.1 | 0.1 | 0.1 | 0.1 | 0 | 0.1 | 0.1 |
| Vanillin | 0.1 | 0.0 | 0.1 | 0.0 | 0.1 | 0.1 | 0.2 | 0.0 |
| Trans-Sabinene hydrate acetate | n.d |  | 0.5 | 1.6 | 0.1 | 0.0 | 0.9 | 2.5 |
