# Supplementary material for: Physiological Conjunction of Allelochemicals and Desert Plants
Source: PLoS One. 2013 Dec 10;8(12):e81580. doi: 10.1371/journal.pone.0081580 (PMC3858270; doi:10.1371/journal.pone.0081580)
Supplement: Table S2 — Changes in the composition of volatiles in organic-solvent extract of A. sieberi with the seasons–only 24 major components are shown. n = 18, n.d. = not detected, 0 represents values less than 0.1. (DOC) [file pone.0081580.s005.doc]

**Table S2.** Changes in the composition of volatiles in organic-solvent extract of *A. sieberi* with the seasons—only 24 major components are shown. n = 18, n.d. = not detected, 0 represents values less than 0.1.

|  | **Autumn** | | **Winter** | | **Spring** | | **Summer** | |
| --- | --- | --- | --- | --- | --- | --- | --- | --- |
| **Compound** | **Relative %** | **SD** | **Relative %** | **SD** | **Relative %** | **SD** | **Relative %** | **SD** |
| 1,8-Cineole | 13.5 | 2.4 | 7.9 | 0.8 | 9.5 | 2.4 | 29.1 | 3.3 |
| Camphor | 12.4 | 2.2 | 8.8 | 0.9 | 8.0 | 1.8 | 28.5 | 3.1 |
| Artemisia alcohol | 8.1 | 2.3 | 5.8 | 1.5 | 9.3 | 3.6 | 13.0 | 2.2 |
| trans-Thujone | 5.6 | 1 | 19.7 | 2.2 | 1.6 | 0.5 | 10.4 | 1.5 |
| Borneol | 4.5 | 1.8 | 2.5 | 0.6 | 5.1 | 1.5 | 19.8 | 3.2 |
| Germacrene D | 4.2 | 1.4 | 3.0 | 0.8 | 4.0 | 1 | 14.4 | 3.5 |
| Camphene | 4.1 | 0.7 | 3.0 | 0.4 | 2.9 | 0.7 | 9.1 | 1.1 |
| para-Cymene | 3.2 | 0.6 | 2.0 | 0.3 | 0.5 | 0.2 | 9.2 | 2.1 |
| Sabinene | 1.7 | 0.9 | 4.9 | 1 | 0.8 | 0.3 | 2.7 | 0.6 |
| α-Pinene | 1.3 | 0.4 | 1.8 | 0.4 | 3.1 | 1.1 | 2.4 | 0.6 |
| cis-Sabinene hydrate | 1.1 | 0.3 | 0.8 | 0.2 | 2.0 | 0.7 | 1.5 | 0.3 |
| trans-Sabinene hydrate | 0.8 | 0.1 | 0.4 | 0.1 | 0.7 | 0.2 | 1.4 | 0.3 |
| Benzoic acid (methyl vanillate) | 0.8 | 0.1 | 0.6 | 0.1 | 0.2 | 0.1 | 1.6 | 0.1 |
| (Z) Methyl jasmonate | 0.6 | 0.1 | 0.8 | 0.1 | 0.4 | 0.2 | 0.7 | 0.1 |
| Pinocarvone | 0.5 | 0.4 | 0.7 | 0.1 | n.d | 0 | 1.9 | 0.3 |
| cis-Thujone | 0.4 | 1.4 | 0.7 | 0.1 | n.d |  | n.d |  |
| Jasmine ketolactone | 0.4 | 0.1 | 0.3 | 0.0 | 0.2 | 0.1 | 0.8 | 0.1 |
| α-Tujene | 0.3 | 0.1 | 0.3 | 0.1 | 0.4 | 0.1 | 0.6 | 0.2 |
| Myrtenol | 0.3 | 0.2 | n.d |  | 0.2 | 0.1 | 1.3 | 0.3 |
| Carvacrol | 0.1 | 0.1 | 0.6 | 0.1 | n.d |  | 0.9 | 0.1 |
| Eugenol | 0.1 | 0.0 | 0.2 | 0.0 | 0.2 | 0.0 | 0.3 | 0.1 |
| Terpinene-4-ol | n.d |  | n.d |  | 0.2 | 0.6 | n.d |  |
| Thuj-3-en-10-al | n.d |  | n.d |  | 0.2 | 0.6 | n.d |  |
| (E) Jasmone | n.d |  | 0.1 | 0.0 | n.d |  | n.d |  |
