# Supplementary material for: Physiological Conjunction of Allelochemicals and Desert Plants
Source: PLoS One. 2013 Dec 10;8(12):e81580. doi: 10.1371/journal.pone.0081580 (PMC3858270; doi:10.1371/journal.pone.0081580)
Supplement: Table S3 — Changes in the composition of volatiles in organic-solvent extract of A. judaica with the seasons–only 17 major components are shown. n = 18, n.d. = not detected, 0 represents values less than 0.1. (DOC) [file pone.0081580.s006.doc]

**Table S3.** Changes in the composition of volatiles in organic-solvent extract of *A. judaica* with the seasons—only 17 major components are shown. n = 18, n.d. = not detected, 0 represents values less than 0.1.

| **Compound** | **Autumn** | | **Winter** | | **Spring** | | **Summer** | |
| --- | --- | --- | --- | --- | --- | --- | --- | --- |
|  | **% of total** | **SD** | **% of total** | **SD** | **% of total** | **SD** | **% of total** | **SD** |
| Artemisia ketone | 35.2 | 1.1 | 35.8 | 1.1 | 40.3 | 1.2 | 33.3 | 0.6 |
| (E) Ethyl cinnamate | 17.1 | 0.6 | 17.0 | 0.6 | 11.4 | 0.6 | 18.3 | 0.5 |
| Davanone | 12.0 | 1.1 | 9.6 | 1.8 | 13.3 | 4.0 | 9.6 | 1.7 |
| Artemisia alcohol | 8.4 | 0.7 | 10.2 | 1.7 | 13.6 | 1.4 | 8.6 | 1.1 |
| Filifolide A | 6.7 | 1.0 | 6.7 | 1.3 | 4.5 | 2.3 | 8.5 | 1.3 |
| (Z) Ethyl cinnamate | 6.5 | 0.5 | 6.8 | 0.6 | 5.1 | 1.4 | 6.4 | 0.4 |
| Piperitone | 4.5 | 0.4 | 3.8 | 0.4 | 2.2 | 0.2 | 5.7 | 0.4 |
| β-Davanone-2-ol | 3.2 | 0.3 | 3.6 | 0.2 | 2.4 | 0.3 | 2.7 | 0.1 |
| Chrysanthenone | 2.0 | 0.2 | 1.8 | 0.3 | 2.4 | 0.4 | 1.6 | 0.2 |
| nor-Davanone | 0.8 | 0.2 | 0.9 | 0.3 | 0.8 | 0.1 | 1.0 | 0.1 |
| Yomogi alcohol | 0.8 | 0.1 | 0.9 | 0.1 | 0.5 | 0.2 | 0.8 | 0.1 |
| Camphor | 0.6 | 0.1 | 0.6 | 0.1 | 0.8 | 0.2 | 0.7 | 0.1 |
| Sabinene | 0.6 | 0.1 | 0.4 | 0.1 | 0.6 | 0.2 | 0.5 | 0.1 |
| Borneol | 0.5 | 0.3 | 0.6 | 0.1 | 0.6 | 0.1 | 1.0 | 0.1 |
| Methyl vanillate | 0.5 | 0.1 | 0.6 | 0.1 | 0.7 | 0.5 | 0.6 | 0.2 |
| Jasmine ketolactone | 0.2 | 0.0 | 0.2 | 0.0 | 0.2 | 0.0 | 0.2 | 0.0 |
| Methyl epi-jasmonate | 0.2 | 0.0 | 0.3 | 0.0 | 0.3 | 0.1 | 0.2 | 0.0 |
