# Supplementary material for: Physiological Conjunction of Allelochemicals and Desert Plants
Source: PLoS One. 2013 Dec 10;8(12):e81580. doi: 10.1371/journal.pone.0081580 (PMC3858270; doi:10.1371/journal.pone.0081580)
Supplement: Table S4 — Eigenvector values (in descending order) of metabolites were calculated by PCA algorithm for 1st and 2nd components of targeted samples in A. judaica (A), A. sieberi (B) and O. dayi (C). (DOC) [file pone.0081580.s007.doc]

**Table S4**. **(A)**

| **Metabolite** | **PC1** | **Metabolite** | **PC2** |
| --- | --- | --- | --- |
| Filifolide A | 2.53694 | Sabinene | 1.99127 |
| Piperitone | 1.97101 | Isobutyl benzene | 1.87037 |
| Yomogi alcohol | 1.72638 | Piperitone | 1.68978 |
| Methyl vanillate | 1.49807 | Davanone | 1.63271 |
| Z Ethyl cinnamate | 1.24845 | E Ethyl cinnamate | 1.53464 |
| E Ethyl cinnamate | 1.16938 | Methyl vanillate | 1.44403 |
| Methyl jasmonate | 0.91309 | β-Davanone-2-ol | 1.37999 |
| nor-Davanone | 0.90662 | Methyl epi-jasmonate | 1.37676 |
| Camphor | 0.90405 | Chrysanthenone | 0.98437 |
| β-Davanone-2-ol | 0.86628 | Borneol | 0.81362 |

**(B)**

| **Metabolite** | **PC1** | **Metabolite** | **PC2** |
| --- | --- | --- | --- |
| Filifolide A | 2.53694 | Sabinene | 1.99127 |
| Piperitone | 1.97101 | Isobutyl benzene | 1.87037 |
| Yomogi alcohol | 1.72638 | Piperitone | 1.68978 |
| Methyl vanillate | 1.49807 | Davanone | 1.63271 |
| Z Ethyl cinnamate | 1.24845 | E Ethyl cinnamate | 1.53464 |
| E Ethyl cinnamate | 1.16938 | Methyl vanillate | 1.44403 |
| Methyl jasmonate | 0.91301 | β-Davanone-2-ol | 1.37999 |
| nor-Davanone | 0.90662 | Methyl epi-jasmonate | 1.37676 |
| Camphor | 0.90405 | Chrysanthenone | 0.98437 |

**(C)**

| **Metabolite** | **PC1** | **Metabolite** | **PC2** |
| --- | --- | --- | --- |
| Camphene | 1.18604 | cis-Sabinene hydrate acetate | 0.82559 |
| Borneol | 1.17745 | Isobutyl benzene | 0.81008 |
| Camphor | 1.06297 | α-Pinene | 0.74949 |
| Terpinene-4-ol | 0.9877 | cis-Sabinene hydrate | 0.6476 |
| Isobutyl benzene | 0.98223 | α-Tujene | 0.61495 |
| Thuj-3-en-10-al | 0.77901 | Eugenol | 0.20377 |
| Artemisia alcohol | 0.56317 | cis-Thujone | 0.17483 |
| cis-Sabinene hydrate | 0.56209 | Carvacrol | 0.01307 |
| Germacrene D | 0.39783 | trans-Sabinene hydrate | 0.00285 |
| E Jasmone | 0.34641 | E Jasmone | -0.38515 |
